# Topical sodium valproate-loaded nanospanlastics versus conventional topical steroid therapy in *alopecia areata*: a randomized controlled study

**DOI:** 10.1007/s00403-023-02785-1

**Published:** 2024-01-03

**Authors:** Rania M. Mogawer, Marwa Mohamed Fawzy, Ahmed Mourad, Heba Ahmed, Maha Nasr, Zeinab Ahmed Nour, Vanessa Hafez

**Affiliations:** 1 Dermatology Department, Kasr Alainy Faculty of Medicine, Cairo University, Cairo, Egypt; 2 Pharmaceutics and Industrial Pharmacy, Faculty of Pharmacy, Ain Shams University, Cairo, Egypt; 3 Biochemistry Department, Kasr Alainy Faculty of Medicine, Cairo University, Cairo, Egypt

**Keywords:** Alopecia areata, Steroids, Sodium valproate, Beta-catenin

## Abstract

**Background:**

A myriad of therapeutic modalities for alopecia areata are available; however, none is of high level of evidence, creating an immense need for the evaluation of other treatment modalities, of which topical sodium valproate is of potential role via proposed decrease in beta-catenin breakdown, despite its well-known side effect of hair fall as an oral therapy.

**Objective:**

Evaluating the efficacy and the safety of sodium valproate (SV)-loaded nanospanlastics, in comparison to topical corticosteroids, this is the currently available gold standard topical treatment for patchy AA.

**Methodology:**

A total of 66 patients with patchy AA were randomly assigned to receive either topical mometasone furoate lotion or topical SV applied twice daily to all patches except a control patch, which was left untreated. Clinical, trichoscopic and biochemical assessments of beta-catenin tissue levels and Axin-2 gene expression were carried out at baseline and after 3 months.

**Results:**

Both therapeutic modalities were comparable. Potential efficacy was highlighted by significant improvement in the representative patch, the largest treated patch, to the control patch, the smallest untreated patch in both steroid and valproate groups (*p* = 0.027, 0.003 respectively). Both beta-catenin levels and Axin-2 gene expression were reduced after treatment, pointing to the inhibitory effect of dominating uncontrolled inflammatory milieu. Baseline beta-catenin was found to significantly negatively correlate with improvement in the representative patch in patients with baseline level above 0.42 ng/ml (*p* = − 0.042).

**Conclusion:**

Both topical SV and steroids are of comparable modest efficacy. Thus, further evaluation of SV is due in combination with intralesional steroids and other anti-inflammatory treatment modalities, together with developing individualized approaches based on baseline beta-catenin level.

**ClinicalTrials.gov Identifier:**

NCT05017454, https://clinicaltrials.gov/ct2/show/NCT05017454.

## Introduction

Alopecia areata (AA) is an auto-immune inflammatory disorder with a tremendous negative impact on patients’ quality of life, psychological status and self-esteem [1]. It has a quite unpredictable prognosis and can run a chronic course with exacerbations and remissions [2], adding to the detrimental psychosocial burden of AA*.*

In spite of the different available options for treatment of AA, none of these modalities have a high level of evidence, with varying responses and frequent relapses [3]. Thus, an urging need for more efficacious safe treatment options has not yet been met.

The Wnt/beta-catenin signaling pathway is a fundamental pathway that regulates both embryonic and adult hair follicle development and growth. Its downstream effects are mediated primarily by the intracellular β-catenin, which translocates into the nucleus and enhances transcription of many genes involved in promoting hair growth [4]. Thus, this pathway stands as a tempting therapeutic target for treating alopecia [5]. Several studies have uncovered the involvement of Wnt/beta-catenin pathway in AA [6–8].

Sodium valproate (SV), the most widely used anticonvulsant, is proposed to inhibit glycogen synthetase kinase-3 beta (GSK-3b) on the neuronal cells, which, in turn decreases the breakdown of beta-catenin upregulating the Wnt//beta-catenin pathway [9]. Hair-promoting effects of SV have been reported in patients with AGA [5, 10].

Thus, this study was designed to scrutinize the potential beneficial effects of SV in AA, using an optimized SV-loaded nanospanlastics topical formulation [5]. The primary aim was assessing the efficacy of sodium valproate-loaded nanospanlastics in the treatment of patchy AA, in comparison to gold standard topical treatment, namely topical steroids, via clinical and trichoscopic evaluation. To further assess the potential effect of SV, biochemical assessment of beta-catenin and Axin-2 gene expression in the lesional scalp of patients with patchy AA was carried out at baseline and after 3-month treatment course in both groups.

## Patients and methods

The current study is a randomized, double-blinded controlled, parallel-group, therapeutic trial that was conducted in the outpatient clinic, Dermatology Department, Cairo University Hospitals. The study protocol has been approved by the scientific committee of Research Ethics Committee, Faculty of Medicine, Cairo University (MD-206-2021), and it was published in clinicaltrials.gov (ClinicalTrials.gov Identifier: NCT05017454, https://clinicaltrials.gov/ct2/show/NCT05017454). An informed written consent for participation and photography was signed by all patients or their guardians. This report followed the CONSORT checklist for reporting of RCTs [11].

Patients of both genders aged ≥ 5 years with patchy AA, defined as less than 50% involvement of the entire scalp [12], having a minimum of 2 patches were recruited. Patients with alopecia totalis, alopecia universalis, ophiasis and those with more than 50% scalp affection as well as those with associated systemic autoimmune or psychiatric disorders and pregnant and lactating females were excluded.

Seventy-five patients were assessed, of which sixty-six met inclusion criteria and were randomized based on a computer-generated list. Allocation concealment was done using sealed opaque envelopes. Patients were randomized to one of two groups:

- Group A: Standard therapy group (Steroid group) which was treated with the marketed mometasone furoate lotion (Borgasone^®^ lotion) that was placed in an anonymized container similar to that of the sodium valproate.
- Group B: Intervention group (sodium valproate nanospanlastics group) was treated with the pharmaceutically prepared optimized SV-loaded nanospanlastics dispersion as described by Badria and colleagues [5].

Both groups applied the treatment twice daily on the affected areas of the scalp for 3 months (end of therapy), apart from a control patch, defined as the smallest patch which was left untreated to evaluate for possible spontaneous remission.

At baseline, patients were evaluated using Severity of Alopecia Tool (SALT) score [12]. In this study, SALT II visual aid was used as it includes smaller increments of scalp coverage [13] which is more accurate in assessment of localized hair loss in patchy AA. Additionally, trichoscopic evaluation was carried out on both the representative, the largest treated patch, and the control patch. Representative and control patches were trichoscopically assessed for both terminal and dystrophic hairs [14] as well as trichoscopic signs of disease activity, which include exclamation mark hairs, black dots, tapering hairs, broken hairs, and short vellus hairs [15, 16] using DermLite HÜDTM handheld dermoscope, attached to a smart phone using magnetic adapter. Trichoscopic parameters for each patch were quantified in the center of the patch and at four representative fields in the periphery designated as 3, 6, 9, and 12 o'clock positions.

By the end of 3 months, patients in both groups were evaluated for response using alopecia areata physician global assessment (PGA) [12], patient global assessment of improvement (PGAI) [12], mean change in the SALT score [12] as well as trichoscopic improvement in grading scale of terminal and dystrophic hairs in both representative and control patches [14].

**Fig. 1:**
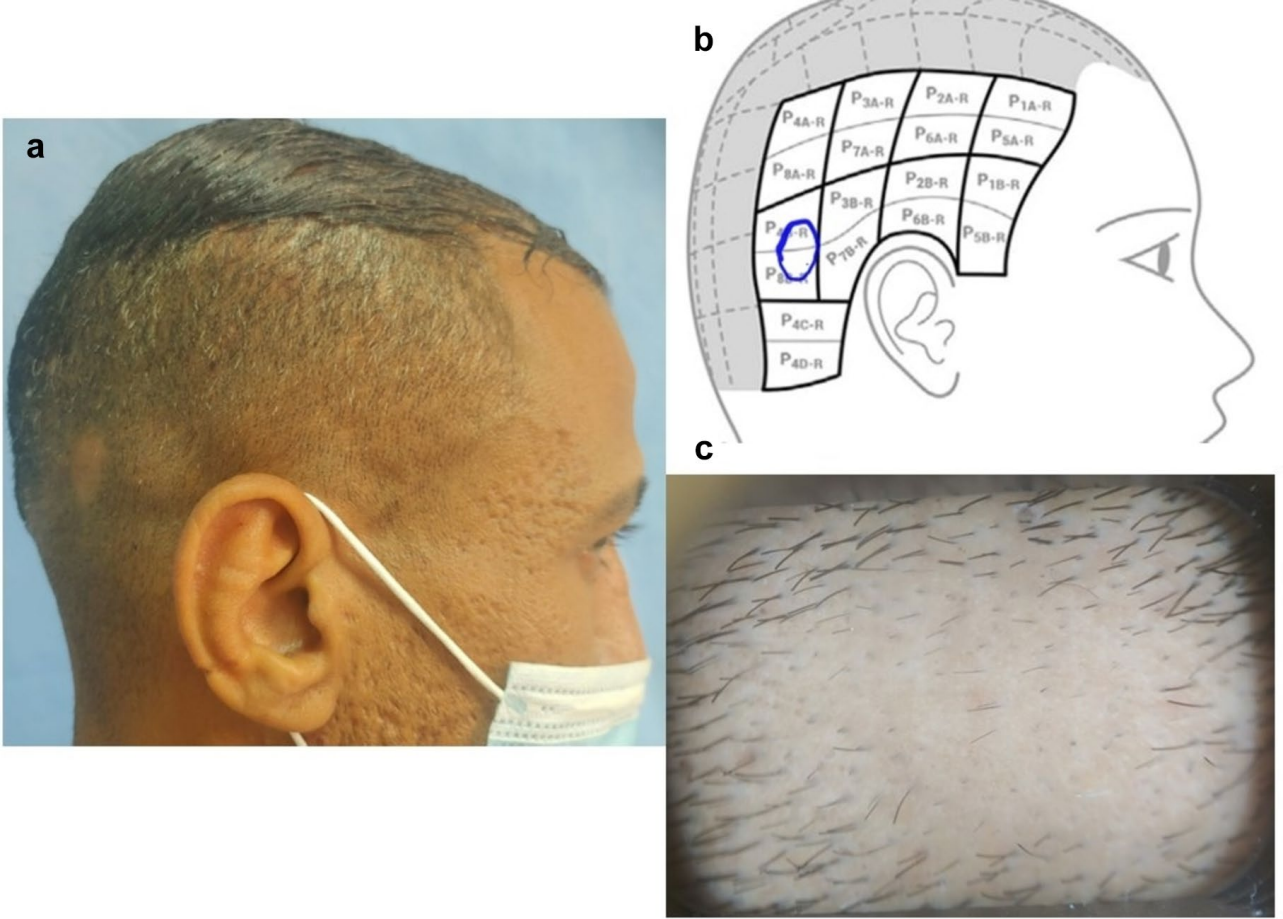
Lesional score calculation. **a** The clinical picture. **b** Lesions sketched on the visual aid tool II. Each unit represents 1% of the scalp surface area. **c** Trichoscopic picture of the lesion. Lesional score = [extent as per visual aid II × trichoscopically assessed hair loss (100-terminal hair density)]/100. Lesional score = [0.5 × 95]/100 = 0.475

A lesional assessment score was implemented by the authors as a modification of lesional area density (LAD) score [17], where extent was expressed in units from 1 to 100 unit as per visual aid II [13]. This was multiplied by hair loss rather than density to reflect disease severity, where a higher score indicates a more severe disease. Hair loss was assessed trichoscopically using a 100-point scale compared with normal (0 = no hair loss to 100 = complete baldness) (hair loss = 100-terminal hair density) (lesional score = [extent × hair loss]/100) (Fig. 1).

All assessments were done by two investigators, one of whom was blinded. Primary outcome was set as treatment success defined as 50% improvement in SALT score and/or PGAI ≥ 50%. Secondary outcomes included improvement as per lesional, trichoscopic scores and PGA as well as patient satisfaction and change in Dermatology Life Quality Index (DLQI) in both groups. Furthermore, changes in tissue expression of beta-catenin and Axin-2 gene expression were evaluated as secondary outcomes. Safety outcomes included any reported irritation, scaling or other adverse events. Given the psychological burden of the disease, DLQI [18] was assessed at baseline and at the end of the study together with patient satisfaction score using 10-point visual analog scale [19]. Patients who achieved 100% reduction in baseline SALT at the end of the study were followed up for 3 months to monitor any relapses (End of study).

In each group, two 2-mm punch skin biopsies were taken from the representative patch before treatment and at the end of therapy in 3-month time in 22 patients in the valproate group and 23 patients in the steroid group, all aged > 14 years. One of the 2 biopsies taken was collected in an empty test tube and the other in phosphate-buffered saline, then both were stored at − 80 °C. ELISA Kit (supplied by ELK Biotech Co.,LTD (China) CAT: ELK1736) and RT-PCR [provided by RT-PCR) AB-4100/A Verso 1-Step QRT-PCR Kit plus ROX, (Germany)] were employed to assess the expression of beta-catenin protein and Axin2 mRNA respectively before and after treatment.

### Pharmaceutical preparation

#### Materials

Span 60^®^ and Cremophor RH 40^®^ were purchased from Sigma Aldrich company, Germany.

1. Preparation of sodium valproate-loaded nanospanlastics:

Span 60^®^, Cremophor RH 40® were dissolved in ethanol at a ratio of 50:50 w/w.

Sodium valproate was added to the ethanolic solution, which was injected dropwise to a preheated aqueous solution followed by stirring till complete evaporation of the ethanol [20].

2. Particle size, polydispersity index and zeta potential measurement of the prepared nanospanlastics:

The properties of the prepared nanospanlastics were measured by Zetasizer device (Malvern, UK). Results revealed that the particle size of the spanlastics was 250 ± 21.3 nm, and the Polydispersity index (PDI) was 0.35 ± 0.12. The charge on the particles was − 19.81 ± 4.23 mV.

### Statistical analysis

Sample size calculation was done using G*Power 3.1.9.2 for the randomized control trial giving a projected sample of 64 patients, 32 in each group. For the tissue assessment to evaluate response to therapy, a projected sample size of 19 in each group was given.

Data were coded and entered using the statistical package SPSS version 21. These data were summarized using mean ± standard deviation in quantitative data and using frequency (count) and relative frequency (percentage) for categorical data. Median and interquartile range (IQR) were added to express the data which were not normalized. Comparisons between variables over time were done using non-parametrical Friedman test and Wilcoxon test. Comparisons between quantitative variables in the 2 studied groups were done using Mann–Whitney test. For comparing categorical data, chi-square (*x*^2^) test was performed. Exact test was used instead when the expected frequency is less than 5. Linear regression was done to adjust for age and disease duration when comparing baseline SALT between the 2 groups. Correlation was done to test linear relations between quantitative variables by spearman correlation coefficient. *p* values less than 0.05 were considered as statistically significant.

All analyses were performed per protocol analysis except for treatment success, it was based on intention-to-treat analysis.

## Results

**Fig. 2:**
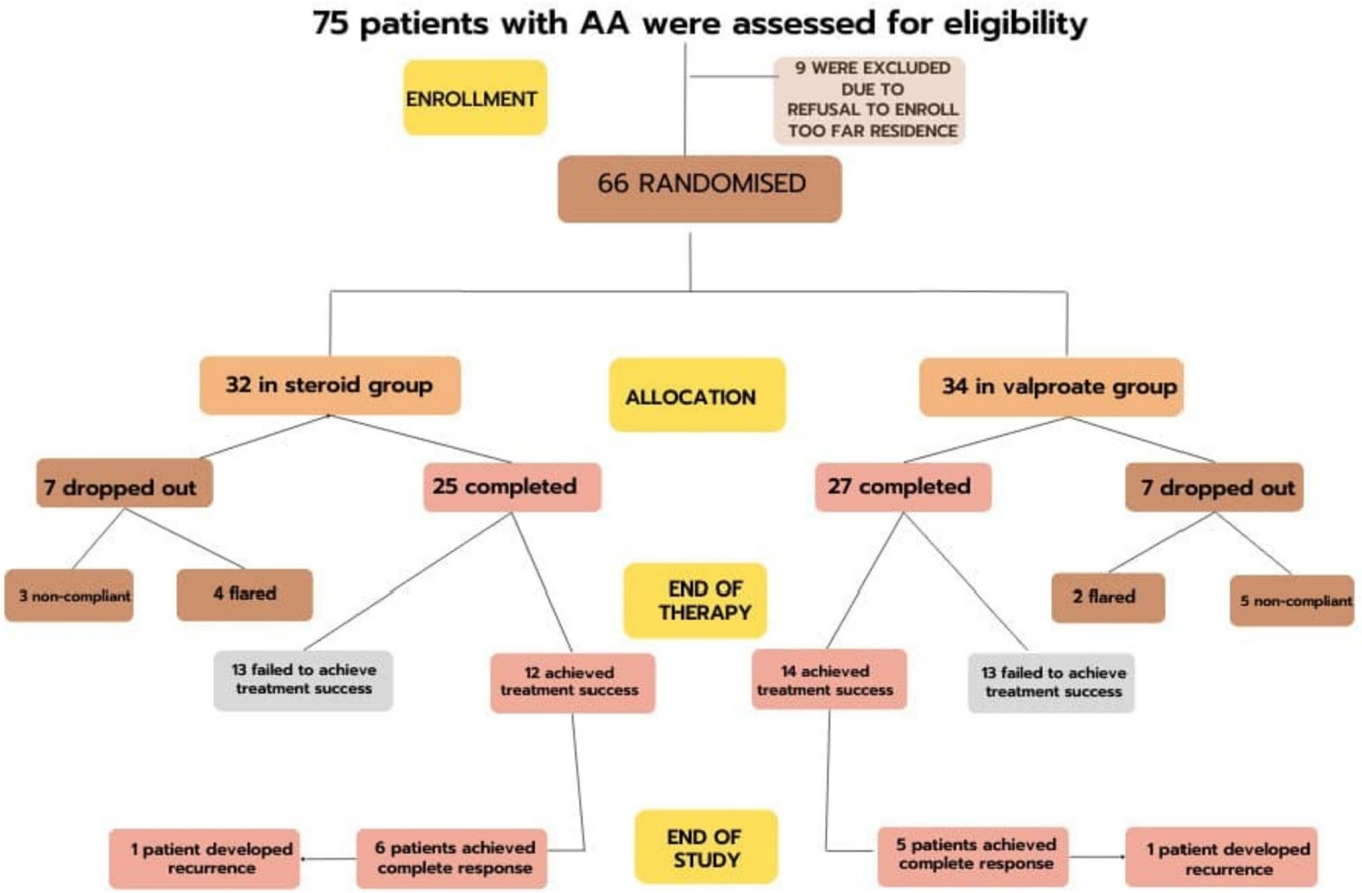
Patients’ flow diagram according to CONSORT guidelines for reporting randomized controlled trials

Out of 75 evaluated patients, 66 were included and randomized to either group **(**Fig. 2**)**. Baseline characteristics of both groups as summarized in Table 1 showed that both groups were homogeneous before treatment.

**Table 1:** Baseline characteristics of steroid group (n = 25) and valproate group (n = 27)

		Steroid Group (n = 32)	Valproate Group (n = 34)	*p* value	(95% CI)
*Age*	Median, (IQR)	31, (22.25)	28, (17.5)	0.763	(− 5 to 7)
*Gender*	M	23 (71.875%)	28 (82.353%)	0.384	(0.49–7.171)
	F	9 (28.125%)	6 (17.647%)		
*Disease duration (months)*	Mean ± SD	14.820 ± 21.523	22.257 ± 51.806	0.602	(− 3 to 1.75)
*Disease activity*	No. (%)	29 (90.6%)	31 (91.2%)	1	(0.278–3.774)
*Positive family history*	No. (%)	2 (6.3%)	5 (14.7%)	0.428	(0.38–28.81)
*Extra-scalp lesions*	No. (%)	6 (18.75%)	5 (14.7%)	0.748	(0.160–3.348)
*BL SALT*	Median, (IQR)	4.813, (4.688)	3.250, (4.250)	0.372	(− 0.75 to 2.5)
*BL lesional score of representative patch*	Median, (IQR)	27, (27)	17.5, (17.938)	0.281	(− 4 to 17)
*BL lesional score of control patch*	Median, (IQR)	9.25, (13.125)	8.875, (7.188)	0.251	(− 1.125 − 5.625)
*BL dystrophic hairs in representative patch*	Median, (IQR)	4, (0)	4, (0)	0.537	(− 9.509 × 10^–6^ − 2.241 × 10^–5)^
*BL terminal hairs in representative patch*	Median, (IQR)	1, (0)	1, (0)	0.779	(− 6.876 × 10–5 − 2.413 × 10–5)
*BL DLQI*	Median, (IQR)	15, (16.5)	18, (13.75)	0.985	(− 5 to 4)

		Steroid Group (n = 17)	Valproate Group (n = 15)	*p* value	95% CI
*Baseline Beta-catenin (ng/ml)*	Mean ± SD	0.519 ± 0.208	0.644 ± 0.222	0.102	(− 0.3–3.578 × 10^–6^)
	Median, (IQR)	0.500, (0.300)	0.620, (0.400)		
*Baseline Axin-2*	Mean ± SD	0.242 ± 0.460	0.187 ± 0.440	0.327	(− 0.012 to 0.094)
	Median, (IQR)	0.063, (0.234)	0.031, (0.055)		

*CI* confidence interval, *IQR* interquartile range, *SD* standard deviation, *SALT* Severity of Alopecia Areata Tool, *DLQI* Dermatology Life Quality Index. *p* value is considered significant if < 0.05, Mann–Whitney *U* test for means, Fisher's exact test for proportions

Fifty-one males (77.273%) and 15 females (22.727%) were included in this study. Their age ranged from 5 to 65 years with a mean age 28.136 ± 13.021, and they were randomly distributed among both groups (*p* = 0.763 and 0.384 respectively).

### Efficacy outcomes

In the steroid group, 13/32 patients versus 17/34 patients in the valproate group achieved treatment success with no statistically significant difference between both groups [95% confidence interval (CI) = − 0.947 to 1.261].

**Fig. 3:**
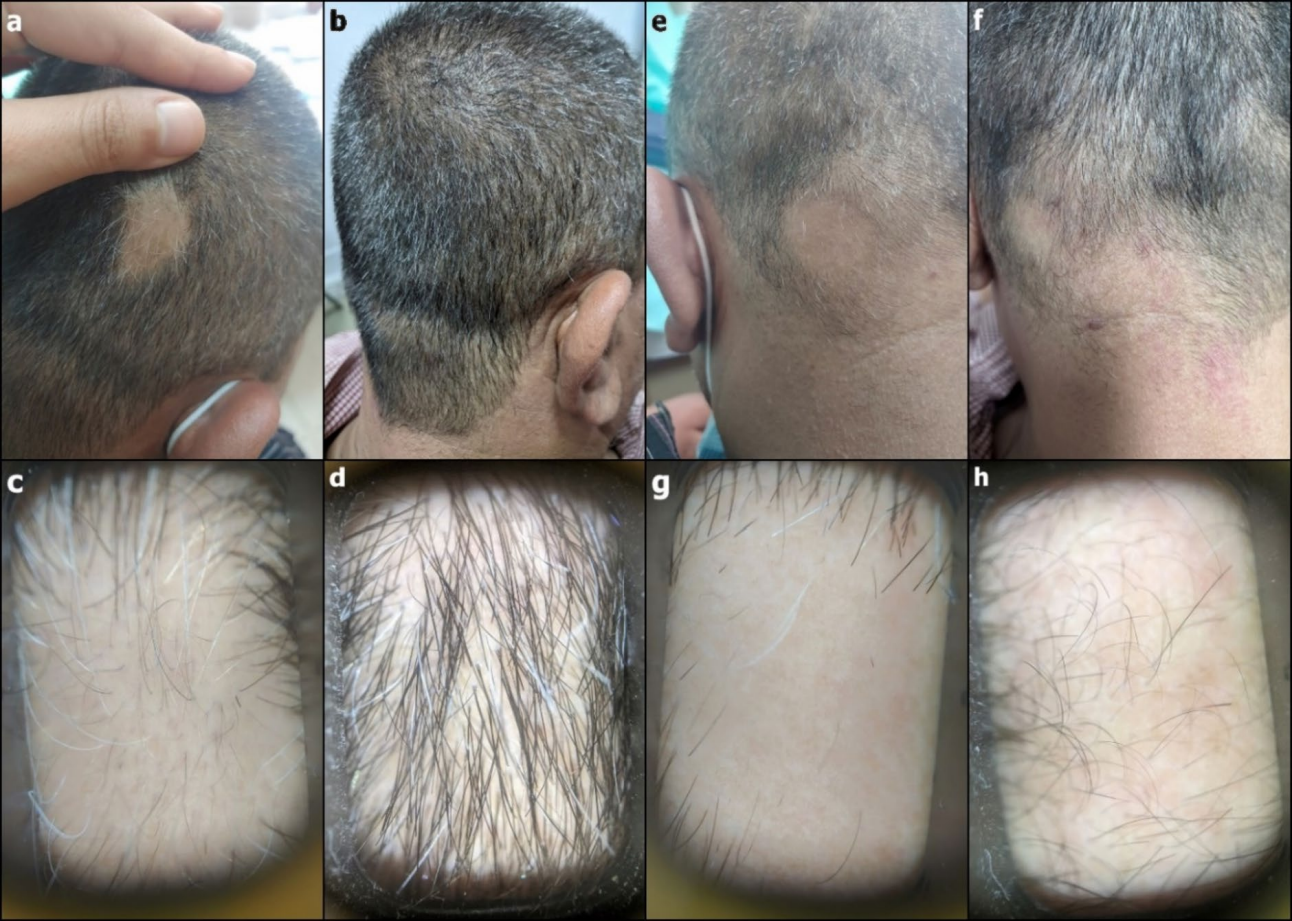
Case showing good response to topical steroid. Representative patch before treatment (**a**) and 3 months after treatment (**b**). Trichoscopy of the representative patch before treatment (**c**) and hair regrowth 3 months after treatment (**d**). Control patch at baseline (**e**) with no improvement was noted after 3 months (**f**). Trichoscopy of the control patch before treatment (**g**) with minimal hair regrowth after 3 months (**h**)

**Fig. 4:**
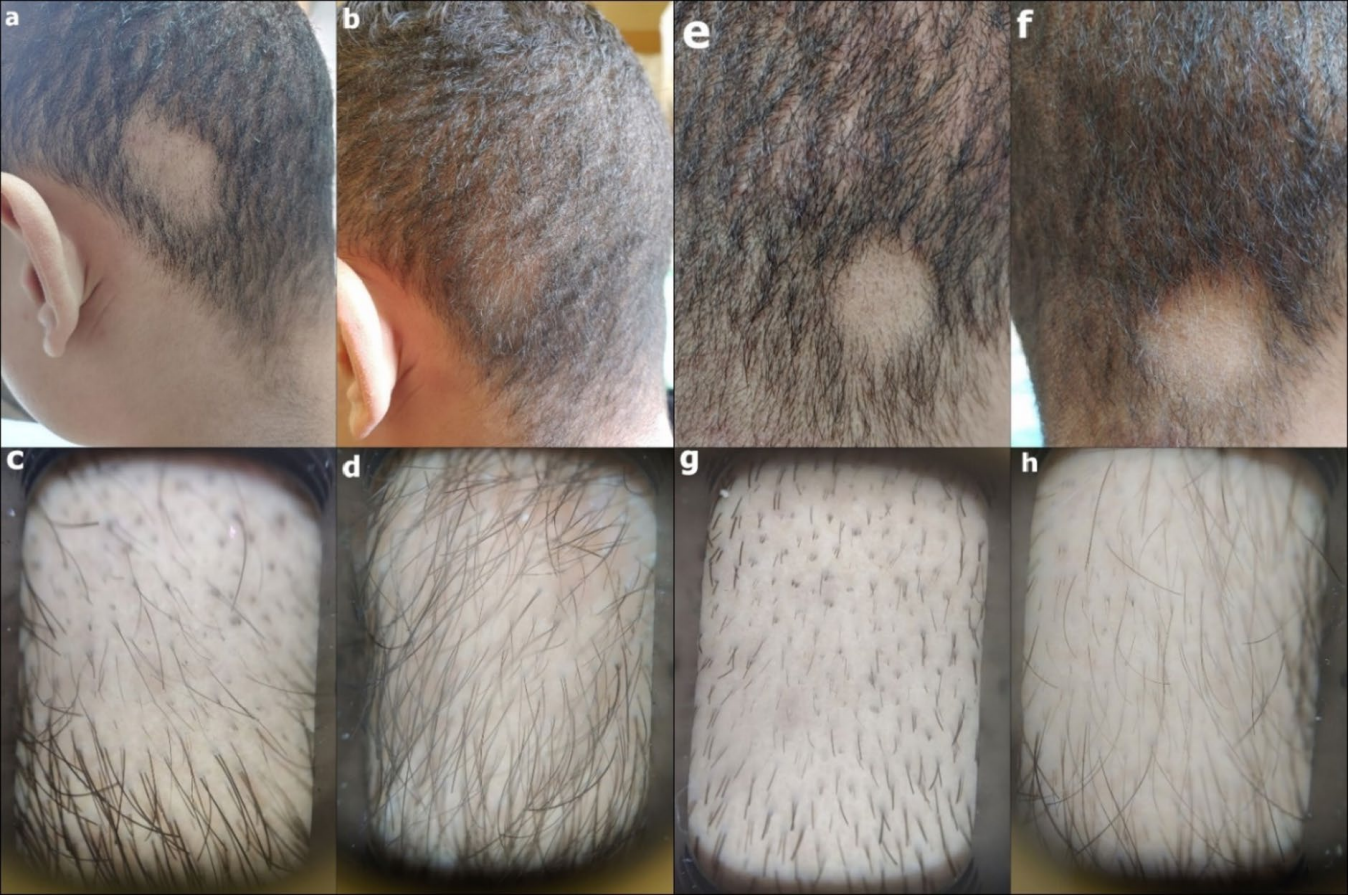
Case showing good response to topical sodium valproate. Representative patch before treatment (**a**) and 3 months after treatment (**b**). Trichoscopy of the representative patch before treatment (**c**) showing hair regrowth 3 months after treatment (**d**). Control patch at baseline (**e**) with no improvement was noted after 3 months (**f**). Trichoscopy of the control patch before treatment (**g**) with minimal hair regrowth after 3 months (**h**)

Patients who completed the study, 27 in the valproate group versus 25 in the steroid group were further assessed for prolonged response of treatment after 3 months of end-of-treatment. There was no significant difference between both groups as regards SALT score, PGA, lesional score, percentage of terminal and dystrophic hair as per trichoscopic analysis as well as DLQI and patient satisfaction (Table 2, Figs. 3, 4).

**Table 2:** Comparison between steroid group (n = 25) and Valproate group (n = 27) at three-month assessment at the end of the therapy (EOT)

	Steroid Group (n = 25)	Valproate Group (n = 27)	*p* value	95% CI
*SALT*				
Median, (IQR)	2, (4)	1, (2.825)	0.623	(− 0.875 to 1.750)
Change from baseline Median, (IQR)	2.5, (3.750)	1.375, (2.188)	0.264	(− 1.175 − 2.625)
*Lesional Score of the representative patch*				
Median, (IQR)	13.625, (28)	5.250, (20.625)	0.14,	(− 1.250–13.250)
Change from baseline Median, (IQR)	7.25, (21)	9.5, (9.75)	0.826	(− 7 to 10)
*PGA*				
Mean ± SD	37.200 ± 30.951	46.944 ± 34.497	0.325	(− 30 to 10)
Median, (IQR)	30, (60)	50, (52)		
*Patient global assessment of Improvement*				
Mean ± SD	1.688 ± 1.635	2.118 ± 1.871	0.393	(− 18.369 × 10^–5)^
Median, (IQR)	1(3)	2(4)		
*DLQI*				
Mean ± SD	21.712 ± 25.475	13.224 ± 16.024	0.766	(− 4 to 3)
Median, (IQR)	4 (20)	4 (14)		
*Patient satisfaction*				
Median, (IQR)	5, (5)	7, (4)	0.370	(− 3 to 1)
*Trichoscopic scores*				
Mean dystrophic hairs ± SD	1.88 ± 1.416	1.963 ± 1.709	0.896	(− 1 to 1)
Median, (IQR)	2, (2)	1.5, (3.75)		
Mean reduction from BL	1.98 ± 1.319	1.796 ± 1.689	0.656	(− 1 to 1)
Median, (IQR)	2, (2)	2, (3.25)		
Mean terminal hairs ± SD	2.86 ± 1.327	1.499 ± 0.288	0.87	(− 5 to 1)
Median, (IQR)	3, (1.5)	3.5, (3)		
Mean increase from BL	1.86 ± 1.186	1.537 ± 1.358	0.444	(− 5 to 1)
Median, (IQR)	2, (1.5)	2, (3)		

	Steroid Group (n = 17)	Valproate Group (n = 15)	*p* value	95% CI
*Beta-catenin (ng/ml)*				
Mean ± SD EOT	0.476 ± 0.156	0.427 ± 0.096	0.48	(0.05–0.1)
Median, (IQR) EOT	0.43, (0.1)	0.4, (0.14)		
Mean change ± SD EOT	− 0.056 ± 0.260	− *0.274* ± *0.216*	0.019	(0.04–0.4)
Median, (IQR) EOT	− 0.05, (0.3)	− 0.255, (0.34)		
*Axin-2 gene expression*				
Mean ± SD EOT	0.027 ± 0.061	0.034 ± 0.044	0.471	(− 0.031–0.004)
Median, (IQR) EOT	0.008, (0.015)	0.016, (0.06)		
Log base 2 of the fold change Mean ± SD	− 4.471 ± 5.375	− 3.533 ± 5.914	0.649	(− 5 to 3)
Median, (IQR)	− 4, (9)	− *2, (4.5)*		

*CI* confidence interval, *SALT* Severity of Alopecia Tool, *IQR* interquartile range, *EOT* end of therapy, *PGA* Physician Global Assessment, *DLQI* Dermatology Life Quality Index, *BL* baseline. *p* value is considered significant if < 0.05, Mann–Whitney *U* test

On biochemical assessment, there was no significant difference in beta-catenin level and Axin-2 gene expression in both groups at baseline (95% CI: − 0.3–3.578 × 10^–6^, − 0.012 to 0.094 respectively). After 3 months of treatment, although we did not find significant difference in the mean values of beta-catenin levels at EOT between both groups (CI = 0.05–0.1), patients in the valproate group demonstrated significant reduction in baseline levels of beta-catenin in comparison to steroid patients (CI = 0.04–0.4). As for Axin-2 gene expression, there was no significant difference between both groups as regards mean values at EOT and fold change in baseline expression (CI = − 0.031 to 0.004, − 5 to 3 respectively) (Tables 1, 2).

The control (the smallest patch that was left untreated) and the representative (the largest treated patch) patches in each group were compared in order to evaluate the possibility of spontaneous remission. In both steroid and valproate groups, improvement in the lesional score of the representative patch (11.449 ± 17.778 and 8.985 ± 12.383 respectively) was significantly higher than the improvement of the control patch (4.625 ± 16.214 and 2.675 ± 7.301 respectively) (*p* = 0.027, 0.003 respectively). A median of 7.25 and an interquartile range (IQR) of 21 for improvement of the representative patch versus a median of 3.875 and IQR of 15.75 for the control patch were reported in the steroid group. In the valproate group, a median of 9.5 and an IQR of 9.75 for improvement of the representative patch versus 4.013 and 5.825 for the control patch were reported.

Baseline disease characteristics were tested for their impact on treatment outcome. None of them influenced clinical outcomes nor laboratory measurements of beta-catenin levels and Axin-2 gene expression.

Additionally, significant correlation was detected between baseline levels of beta-catenin and its change after treatment (*p* < 0.001), and a linear regression model was developed to explain this finding. It showed that every one-unit increase in baseline beta-catenin is associated with 0.965-unit decrease in the change of the beta-catenin from the baseline.

$${\text{Predicted change in beta - catenin}} = - 0.965 \times {\text{baseline beta - catenin}} + 0.418.$$

**Fig. 5:**
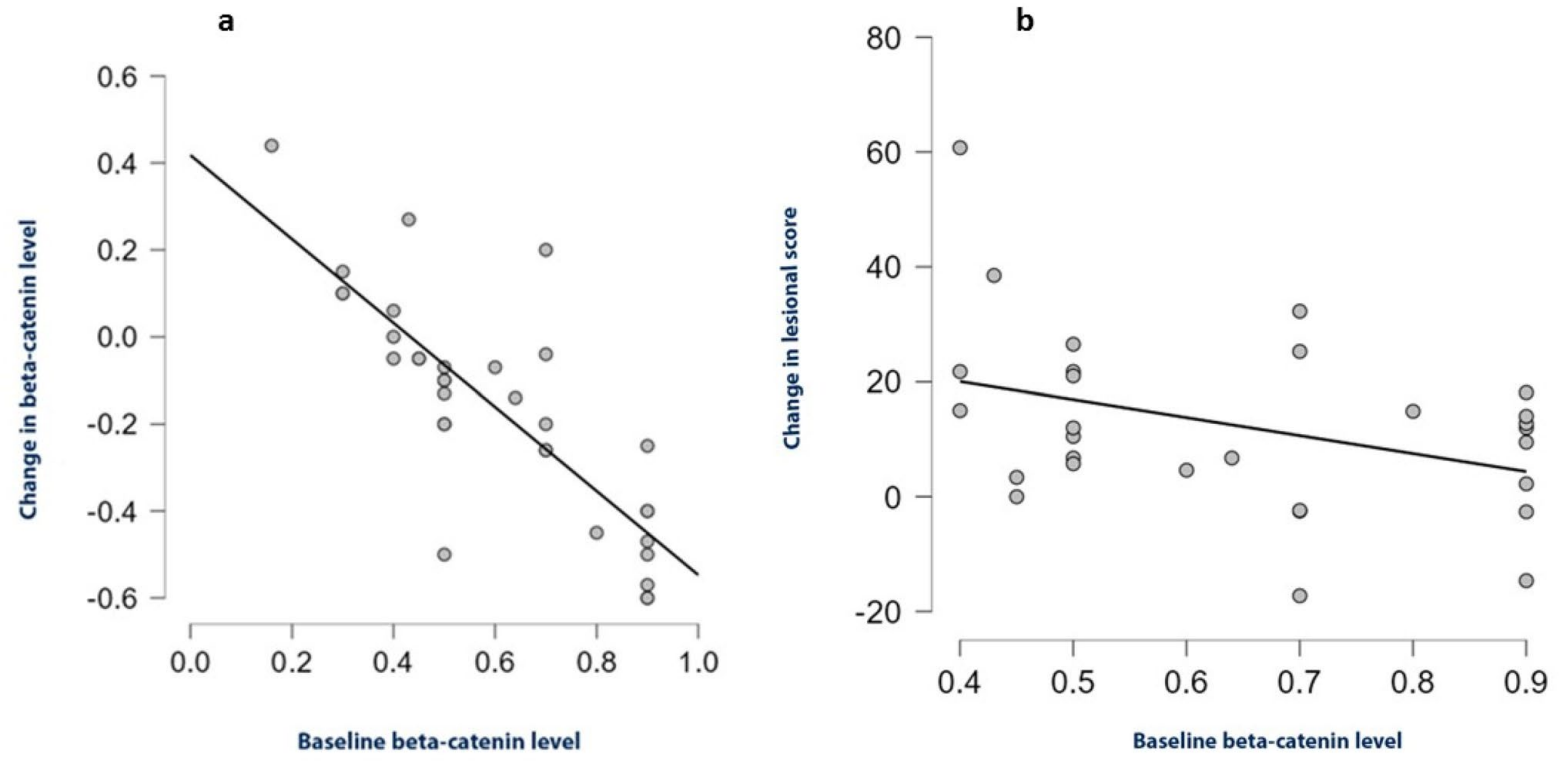
**a** Scatterplot and line of best fit for baseline beta-catenin levels and its change after treatment (intercept = 0.418, slope = − 0.965, Adjusted *R*-squared: 0.650, *p* value < 0.001). **b** Scatterplot and line of best fit for baseline B catenin levels and change in lesional score after treatment

When baseline beta-catenin level was 0.42, our model predicted no change in beta-catenin, above which it decreased with therapy and below which it increased (Fig. 5a).

Mild negative correlation was detected between the change in lesional score of the representative patch after treatment and baseline beta-catenin levels in patients whose baseline beta-catenin was above 0.42 ng/ml (*p*—0.042) **(**Fig. 5b**)**.

### Lesional Score

Lesional score: a proposed modification to the LAD score [17], correlated positively and significantly with SALT at baseline (rho = 0.682, *p* < 0.001) and end of treatment (rho = 0.607, *p* < 0.001) as well as their change on treatment (rho = 0.799, *p* < 0.001); however, median change in the lesional score was more pronounced.

In steroid group, relapse was reported in 1/6 (8.33%) versus 1/5 (11.54%) in valproate group, with no significant difference between both groups (*p* = 1).

Both treatment modalities were tolerated with good safety profile, without significant difference in the reported adverse events (*p* = 0.67). In valproate group, only 4 patients (14.82%) reported mild scaling, while in steroid group, two patients (8%) reported side effects; namely, mild scaling in one patient, and folliculitis in another patient.

## Discussion

Alopecia areata (AA) is quite difficult to treat, and only few treatments have been evaluated in randomized controlled trials. Intralesional and topical steroids are the gold standard therapy for limited patchy disease. Other topical agents include topical minoxidil, prostaglandin analogs, contact immunotherapy and topical JAK inhibitors [3]. As an attempt to expand the topical AA therapeutic armamentarium, we aimed to evaluate the efficacy and safety of topical sodium valproate in AA.

Sixty-six patients with mild to moderate AA were randomly assigned to receive either mometasone furoate lotion or topical sodium valproate lotion twice daily for a total duration of 3 months. Treatment success was defined as 50% improvement in SALT and/or PGAI. The proportion of patients who achieved successful treatment was slightly higher in the valproate group (50%) than the steroid group (40%). Yet, both treatments were comparable in efficacy with no significant difference.

These results are in line with previous studies, showing modest efficacy of topical corticosteroids. Devi and colleagues (2015) compared the efficacy of intralesional steroids with betamethasone valerate 0.1% cream in 226 patients with localized AA, they reported adequate hair regrowth in less than half of patients in the topical steroid group [21]. Additionally, similar therapeutic response was obtained in a study that compared topical mometasone furoate cream to bimatoprost 0.03% solution in 60 patients with limited AA [22].

An optimized formulation of sodium valproate-loaded nanospanlastics has recently been assessed and proven effective in the treatment of androgenetic alopecia (AGA) [5], which is postulated to act through upregulation of the Wnt/beta-catenin signaling pathway via inhibition of GSK-3b, in simulation to its effect on the neuronal cells [19]. The Wnt/beta-catenin pathway is fundamental for hair follicle development and growth. Its downstream effects are mediated primarily by the intracellular molecule beta-catenin, which translocates into the nucleus and enhances transcription of many genes involved in promoting hair growth [4].

In AA, the Wnt/beta-catenin signaling pathway was found to be suppressed [6–8], rendering this intricate pathway a potential therapeutic target in AA research landscape. In our study, sodium valproate-loaded nanospanlastics formulation was evaluated in the treatment of AA for the first time in comparison to topical steroid in order to scrutinize its reported hair promoting effects via proposed upregulation of the Wnt/beta-catenin pathway.

In a trial to assess patients more objectively, we developed the lesional score. It offers a simple semi-objective tool for assessing alopecia, that is better suited to small lesions using the modified SALT visual aid II [13], while taking into account the hair density, unlike the original salt score that considered only alopecic patches that were devoid of terminal hair [17].

Efficacy of both treatment modalities was further highlighted by the significant improvement from baseline in SALT, lesional and trichoscopic scores by the end of 3-month treatment course as well as the observed significant improvement from baseline in the representative patch in comparison to the control patch. This sheds light on the potential role of SV in treatment of AA.

It is noteworthy that the control patch has shown some improvement which was slightly higher in the steroid group than the valproate group. This might denote higher, yet insignificant, incidence of spontaneous remission in the steroid group. However, possible diffusion and lack of abidance of the patients with strict avoidance of treatment application to the control patch are possible hypotheses. Two patients having AGA have reported improvement of their hair density during their course of treatment with SV; thus, diffusion of the applied treatment is plausible, but needs further evaluation.

In our study, we followed up patients who achieved complete resolution for additional 3 months to monitor for any relapses. In fact, most of them were able to maintain the attained response apart from one patient in each group. Interestingly enough, the relapsing patient in the valproate group had the hair loss exactly in the site of previously treated patches, which reversed upon reintroduction of topical SV.

Accumulating body of evidence uncovered disrupted Wnt signaling in AA. Lim et al. reported reduced expression of beta-catenin in AA patients in comparison to controls [6]. Additionally, Dickkopf 1 (DKK-1), a powerful suppressor of the Wnt/β-catenin signaling pathway, was found to be significantly higher in tissue of patients with AA compared to controls [7, 8].

Furthermore, IFN-γ, which is a fundamental cytokine in AA pathogenesis, was found to induce catagen-like changes in human dermal papilla cell (HDPC) culture and in hair follicles via inhibition of Wnt/β-catenin signaling, mainly by increasing DKK-1 expression and activating GSK3-b [23].

In our study, we detected reduction in beta-catenin levels and Axin-2 gene expression in both steroid and valproate group after 3 months of therapy, with this reduction being more significant in valproate group in comparison to steroid group. We were intrigued by this finding as this contradicts what was postulated, where sodium valproate was suggested as a potential up-regulator of the Wnt/beta-catenin signaling pathway via decreasing the breakdown of beta-catenin [9].

As an attempt to justify this interesting finding, we proposed the following speculations. Primarily, it's noteworthy that SV was found to increase beta-catenin in murine models and in vitro HDPC cultures [24, 25]. This could be altered in hair disorders that affect hair cycling dynamics such as AA.

Second, given that the majority of our patients in both groups were active as per history and trichoscopic analysis, this reflects the underlying inflammatory milieu dominated by IFN-γ that turns off Wnt signaling through increasing DDK-1 expression and eventually increasing beta-catenin degradation and depleting its cytosolic pool. Therefore, by considering that SV was reported to inhibit GSK3-b and decrease beta-catenin degradation and not increasing its synthesis [25], this could in part explain why beta-catenin levels did not increase after treatment as SV action is antagonized by the over-expressed DKK-1 that depletes the cytosolic pool of beta-catenin.

In addition, we noted that in cases who applied topical steroids (whose depth of penetration in comparison to intralesional steroids is quite limited), the drop in beta-catenin was insignificant in comparison to sodium valproate group. Thus, we hypothesize that a higher inflammatory milieu in the valproate group, which is unlikely to be targeted by SV, is a possible cause for the significant reduction in the beta-catenin in this group.

Since we could not verify the proposed hair promoting effect of SV via upregulating beta-catenin expression, we suggest that the reported therapeutic response of SV in our study might be attributed to its potential effects on other pathways including protein kinase C, and extracellular signal-regulated kinase (ERK) [9, 26], which needs further evaluation in future studies.

This drives us to the assumption that the priority in treatment of AA is targeting the ongoing autoimmune inflammatory process that inhibits the Wnt/beta-catenin. Thus, SV needs to be re-evaluated in synergy with steroids, in simulation to the use of minoxidil with steroids in treatment of AA, which was found to upregulate the Wnt/beta-catenin pathway [27]. Additionally, the modest efficacy of topical steroids as well as failure to improve the beta-catenin expression may highlight the superior efficacy of intralesional steroids as the first line treatment of limited AA, whenever tolerated.

Different baseline disease characteristics, such as duration and severity of AA, were not of influence on the outcome of treatment. However, baseline beta-catenin was found to correlate inversely and significantly with the change in beta-catenin in all patients. Both SV and steroids were hypothesized to increase beta-catenin level via inhibiting GSK3-b [9, 28], which is responsible for beta-catenin breakdown, rather than increasing its production [4]. Therefore, the treatment is predicted to increase beta-catenin only to its baseline production capacity, which is speculated to be around 0.42 by our model, under which beta-catenin is readily increased by limiting its destruction. Yet, above this level, baseline beta-catenin gradually reduced by the ongoing active disease.

It is worth mentioning that the subgroup of patients, exceeding this threshold level, had a significant negative correlation between the baseline beta-catenin level and with the reduction in the lesional score. In other terms, higher baseline beta-catenin seems to be correlated with poorer response to treatment. Accordingly, we hypothesize that the mechanistic underpinning of SV is better suited to patients with low baseline beta-catenin, while patients with high baseline beta-catenin are more likely to benefit from anti-inflammatory drugs. This, in turn, opens the door for developing personalized treatment regimens and further research on the patient subgroups that are better poised to benefit from such therapy.

Lesional score was found to correlate positively with SALT score; however, the median change in the lesional score was more pronounced than in the SALT in both groups, highlighting the greater ability of the lesional score to reflect minute changes in hair growth.

Both treatments were well tolerated in both groups. However, mild scaling on SV group was documented in around 15% of the patients, and this is in accordance with what Badria and colleagues reported [5]. The notable scaling with SV may imply that topical SV induces GSK-3b inhibition and activation of Wnt/beta-catenin pathway not only in the hair follicles but in the epidermis as well, therefore enhancing keratinocyte proliferation that manifests clinically as scaling [29].

This study is limited by lack of data about beta-catenin expression in non-lesional scalp to limit the number of tissue biopsies from each patient.

In conclusion, sodium valproate was found of comparable efficacy to topical steroids. However, better outcomes are opted for, which were not achievable by either treatment modalities. The postulated mechanism of action of sodium valproate through upregulation of Wnt/beta-catenin signaling pathway could not be verified in this study, and further research is needed to explain this surprising result. The lesional score suggested by the authors in this work might be of interest to researchers who assess treatments on alopecia areata for more accurate assessment of individual response of lesions.

## Data Availability

Participants' data that undelie reported results will be shared upon request, after deidentification, for researches who provide a methodilogically sound proposal to access data to achieve aims in the approved proposal. This is beginning 3 months up to 12 months after publication.
